# The complete mitochondrial genome of Malayan Gaur (*Bos gaurus hubbacki*) from Peninsular Malaysia

**DOI:** 10.1080/23802359.2019.1640085

**Published:** 2019-07-13

**Authors:** Norsyamimi Rosli, Frankie Thomas Sitam, Jeffrine Japning Rovie-Ryan, Han Ming Gan, Yin Peng Lee, Hartini Ithnin, Millawati Gani, Mohd Firdaus Ariff Abdul Razak, Badrul Munir Md-Zain, Mohd Tajuddin Abdullah

**Affiliations:** a National Wildlife Forensic Laboratory (NWFL), Department of Wildlife and National Parks, Kuala Lumpur, Malaysia;; b Institute of Tropical Biodiversity and Sustainable Development, Faculty of Science and Technology, Universiti Malaysia Terengganu, Terengganu, Malaysia;; c Department of Genomics Facility, Monash University Malaysia, Selangor, Malaysia;; d School of Life & Environmental Sciences, Faculty of Science, Engineering and Built Environment, Deakin University, Australia;; e School of Environment and Natural Resource Sciences, Faculty of Sciences and Technology, Universiti Kebangsaan Malaysia, Selangor, Malaysia;; f School of Marine and Environmental Sciences, Universiti Malaysia Terengganu, Terengganu, Malaysia

**Keywords:** Malayan gaur, *de novo* assembly, mitogenome, next-generation sequencing

## Abstract

Here, we present the first complete mitochondrial genome of Malayan Gaur (*Bos gaurus hubbacki*) inferred using next-generation sequencing. The mitogenome is 16,367 bp in length with the structural organization of a typical bovine mitochondrial arrangement comprising 13 protein-coding genes, 21 tRNAs, and 2 rRNAs. No internal stop codon was found in the protein-coding genes. Phylogenetic tree analysis revealed that Malayan gaur is more closely related to Burmese banteng instead of gaur.

## Introduction

Traditionally three subspecies have been recognized based on coloration and size (Duckworth et al. 2016): *Bos gaurus gaurus*; *B. g. readei*; and *B. g. hubbacki*. However, only two subspecies are recognized by the International Union for Conservation of Nature (IUCN) which were based on the measurements of skulls and horns (Groves and Grubb 2011; Duckworth et al. 2016). These two subspecies are *B. g. gaurus* which can be found in India, Nepal, and Bhutan; and *B. g. laosiensis* found in Myanmar, southern China, Laos, Vietnam, Cambodia, Thailand, and Peninsular Malaysia). Nonetheless, this dual classification is still inconclusive as the morphological measurements are based only on a few skull samples, and there is no supporting genetic analysis (Hassanin 2014).

In the past, wild gaur can be found in significant numbers in the states of Pahang, Perak, Kelantan, and Terengganu (Conry 1989). Nonetheless, due to poaching and habitat destruction, recently it is estimated that only around 270–300 individuals of wild gaur still exist in Peninsular Malaysia (DWNP 2012). Here, we present the first complete mitochondrial genome of *B. gaurus* isolate from Peninsular Malaysia with 16,367 bp in length. The full mitogenome reported here have the structural organization of a typical bovine mitochondrial arrangement comprising of 13 protein-coding genes, 21 tRNAs, and 2 rRNAs. No internal stop codon was found in the protein-coding genes. This annotated mitogenome has been deposited in GenBank under accession number MK770201.

## Genomic DNA isolation and mitogenome sequencing

Tissue sample of a deceased male Malayan gaur with studbook number S0029 from Sungkai Wildlife Conservation Centre (Latitude: 4.034714 | Longitude: 101.369063) was used for extraction and analysis to generate the data presented here. The tissue samples are catalogued as WGRB-BGH40 and deposited in the Wildlife Genetic Resource Bank (WGRB) at National Wildlife Forensic Laboratory, Department of Wildlife and National Parks Peninsular Malaysia. Genomic DNA (gDNA) was used to generate sequences library of paired-end, 250-bp reads. It generated 2,541,912 of short-read sequences (SRS) totaling 635,478,000 bases of DNA.

## Assembly and gene annotation

The raw SRS were screened using FastQC (Andrews 2010) and trimmed using BBduk (Bushnell 2015). Trimmed SRS were then assembled using repeated referenced mapping and *de novo* assembly. Repeated reference mapping was performed by employing end-to-end mapping and local mapping using BowTie2 plugin on Geneious Prime software (Biomatters, New Zealand) with high-sensitivity setting and re-iteration using a published mitochondrial genome of gaur as reference (JN632604). A total of 5782 contigs were assembled. NOVOPlasty (Dierckxsens et al. 2017) was used to perform *de novo* assembly of the trimmed SRS. Pair-wise alignment of sequences assembled from repeated referenced mapping and *de novo* were carried out in Geneious Prime and the consensus sequence from the alignment was selected as final sequence. Annotation generated by MITOS Webserver (Bernt et al. 2013) was used to identify protein-coding genes, ribosomal and transfer RNA gene and the result was compared with JN632604.

**Figure 1.**
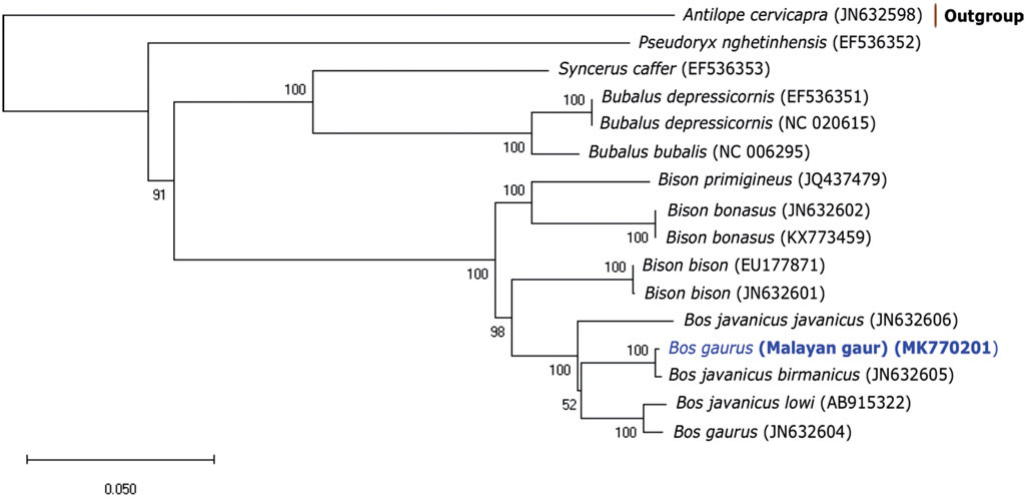
Phylogenetic tree of complete mitogenome among members of Bovini tribe and a member of Antilopini as outgroup inferred using Maximum Likelihood method based on General Time Reversible model with 1000 bootstrap value. GenBank accession numbers for each mitogenomic sequences are shown in parentheses.

Phylogenetic tree (Figure 1) was constructed based on Maximum Likelihood by General Time Reversible model (Nei and Kumar 2000) with 1000 bootstrap value using MegaX software (Kumar et al. 2018). It showed that the Malayan Gaur was grouped together with the Burmese Banteng (JN632605) while the Burmese Gaur (JN632604) emerged as a sister group with the Borneon Banteng (Ishige et al. 2015). However, this grouping is only supported by 52% bootstrap value. More representatives from these two species may help to verify this grouping.
